# Biosensing Tacrolimus in Human Whole Blood by Using a Drug Receptor Fused to the Emerald Green Fluorescent Protein

**DOI:** 10.1021/acs.analchem.2c03122

**Published:** 2022-11-16

**Authors:** Bettina Glahn-Martínez, Giacomo Lucchesi, Fernando Pradanas-González, Ana Isabel Manzano, Ángeles Canales, Gabriella Caminati, Elena Benito-Peña, María C. Moreno-Bondi

**Affiliations:** † Department of Analytical Chemistry, Faculty of Chemistry, Universidad Complutense de Madrid, Plaza de las Ciencias, Ciudad Universitaria, 28040Madrid, Spain; ‡ Department of Chemistry “Ugo Schiff” and CSGI, University of Florence, Via della Lastruccia 13, 50019Sesto Fiorentino, Italy; § Department of Organic Chemistry, Faculty of Chemistry, Universidad Complutense de Madrid, Plaza de las Ciencias, Ciudad Universitaria, 28040Madrid, Spain

## Abstract

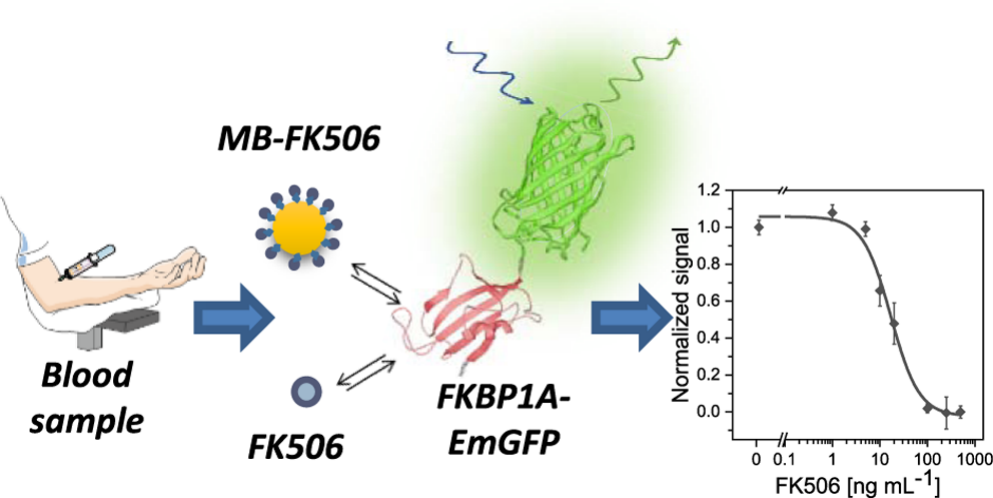

Tacrolimus (FK506) is an immunosuppressant drug (ISD) used to prevent organ rejection after transplantation that exhibits a narrow therapeutic window and is subject to wide inter- and intra-individual pharmacokinetic fluctuations requiring careful monitoring. The immunosuppressive capacity of FK506 arises from the formation of a complex with immunophilin FKBP1A. This paper describes the use of FKBP1A as an alternative to common antibodies for biosensing purposes. Bioassays use recombinant FKBP1A fused to the emerald green fluorescent protein (FKBP1A–EmGFP). Samples containing the immunosuppressant are incubated with the recombinant protein, and free FKBP1A–EmGFP is captured by magnetic beads functionalized with FK506 to generate a fluorescence signal. Recombinant receptor–drug interaction is evaluated by using a quartz crystal microbalance and nuclear magnetic resonance. The limit of detection (3 ng mL^–1^) and dynamic range thus obtained (5–70 ng mL^–1^) fulfill therapeutic requirements. The assay is selective for other ISD usually coadministered with FK506 and allows the drug to be determined in human whole blood samples from organ transplant patients with results comparing favorably with those of an external laboratory.

## Introduction

Tacrolimus (FK506) is one of the most widely used immunosuppressant drugs (ISDs) against organ rejection after transplantation. This compound is a hydrophobic macrolide antibiotic possessing a 23-membered lactone ring (Figure S1) and typically isolated from the bacterium *Streptomyces tsukubaensis*.^1^ FK506 exhibits high inter- and intra-patient pharmacokinetic variability, which, together with its narrow therapeutic window, makes therapeutic drug monitoring absolutely mandatory to adjust and maintain appropriate doses.^2^ Maximizing the efficacy of FK506 while minimizing its toxicity requires its blood concentration levels in transplant recipients to fall within the range 5–20 ng mL^–1^.^3^

Liquid chromatography coupled to tandem mass spectrometry (MS) detection is the currently preferred choice for determining FK506 in clinical laboratories.^4−7^ Although this technique provides accurate, reproducible results, it requires sizeable investments and skilled personnel. In addition, it involves long analysis times and time-consuming sample preparation procedures, which result in increased costs and make the hyphenated technique unsuitable for a high-throughput screening. Immunoassays provide a faster, more economical choice for quantifying FK506. A number of commercial assays for this purpose are currently available, including the cloned enzyme donor immunoassay (Thermo Scientific),^8^ enzyme-linked immunosorbent assay (ELISA),^9^ electrochemiluminescence enzyme immunoassays (ECLIA, Roche Elecsys),^10^ and enzyme-multiplied immunoassay (Siemens)^11^ (Table S5). However, immunoassays are also subject to certain shortcomings such as a limited availability of antibodies against tacrolimus owing to its toxic character, antibody cross-reactivity (CR), and variability between the batches.^12^ Also, although antibody-based assays have been the cornerstone for a number of screening methods, they require using laboratory animals to obtain the antibodies, which is to be avoided according to the recent recommendations of European authorities for protecting the animals used for scientific purposes.^13−15^ One alternative circumventing the ethical issues arising from the use of animal-derived antibodies for bioassay development relies on recombinant proteins with a high specificity and affinity for a given target. Protein engineering techniques have considerably increased the batch-to-batch reproducibility of these biomolecules and enabled their cost-effective large-scale production. In humans, FK506 is recognized by the immunophilin FK506 binding protein 1A (FKBP1A), with which it forms a complex that inhibits calcineurin affecting T-cell activation and proliferation.^16^ Using FKBP1A—which exhibits good specificity and affinity for the immunosuppressant—as a selective recognition element can provide an effective alternative to commonly used antibodies for the development of FK506 biosensors and bioassays. FKBP1A has been used both in native form and genetically modified with a fluorescent protein to examine protein–protein interactions and cell labeling^17−23^ and also to evaluate new ligand substitutes for FK506.^24−28^ Although FKBP1A has additionally been used to develop some sensing platforms,^29−33^ it has never been fused to fluorescent proteins as an alternative to antibodies for developing FK506 quantification bioassays. As in antibody-based commercial assays (ELISA, ECLIA), this approach dispenses with the need for additional reagents to quantify the binding event.

**Figure 1:**
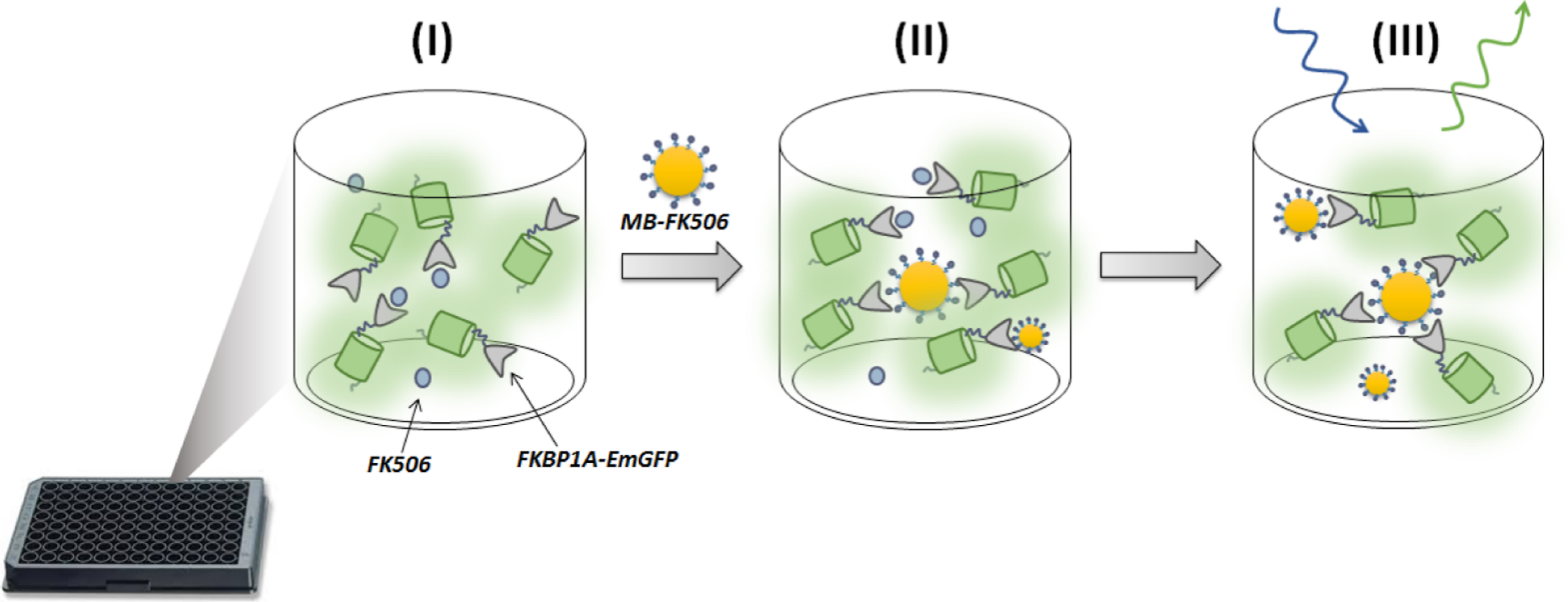
Scheme of the fluororeceptor-based assay for the detection of FK506. (I) The immunosuppressant is recognized by the FKBP1A–EmGFP recombinant protein. (II) Magnetic beads functionalized with FK506 are used to capture free FKBP1A–EmGFP. (III) FKBP1A–EmGFP fluorescence captured by FK506-functionalized beads is monitored following washing.

In this work, we developed a bioluminescent assay to quantify FK506 in whole blood by using a natural receptor of the drug genetically labeled with the emerald green fluorescent protein (EmGFP) as the recognition element. EmGFP was chosen on the grounds of its good photostability and fluorescent properties.^34^ In the assay, the target immunosuppressant is recognized by the FKBP1A–EmGFP-fused protein. After incubation, the magnetic beads functionalized with FK506 (MB-FK506) are used to capture free FKBP1A–EmGFP, which emits a fluorescent signal (Figure 1). The operating conditions of the assay were optimized in terms of protein concentration, buffer composition, and incubation time and temperature. The suitability of the proposed assay was assessed by analyzing whole blood samples from transplant patients under treatment with the immunosuppressant. The results were validated by comparison with those of an external laboratory using a chemiluminescent microparticle immunoassay (CMIA). The proposed assay shows promise for the development of sensitive antibody-free FK506 detection systems.

## Materials and Methods

### Materials

Invitrogen Dynabeads M-270 amine (MB), 2-(*N*-morpholino)ethanesulfonic acid (MES), rapamycin (Sir), Phusion Hot Start II DNA polymerase, chemically competent *E. coli* One Shot BL21 Star (DE3) cells, isopropyl β-d-1-thiogalactopyranoside (IPTG), pRSET-EmGFP plasmid, Sterilin Black Microtiter Plates, and Tween 20 (T20) were purchased from Thermo Fisher Scientific (Rockford, IL, USA). DNA primers were supplied by Integrated DNA Technologies (San Diego, CA, USA). NEBuilder HiFi DNA Assembly Master Mix and NEB 5-alpha competent *E. coli* were obtained from New England BioLabs (Ipswich, MA, USA). *N*-Hydroxysulfosuccinimide sodium salt (sulfo-NHS) and *N*,*N*′-diethylcarbodiimide hydrochloride (EDC) were supplied by Fluorochem (Hadfield, Derbyshire, UK). FKBP1A-pDONR221 plasmid was purchased from DNASU (Tempe, AZ, USA) and tacrolimus (FK506) from Sinoway Industrial (Xiamen, China). Mycophenolic acid (MPA) was from Alfa Aesar (Karlsruhe, Germany). Kanamycin and phosphate-buffered saline with 0.05% T20 (PBST, pH 7.4) were purchased from Sigma-Aldrich (St. Louis, MO, USA). Dimethyl sulfoxide (DMSO) was from VWR (Radnor, PA,USA), sodium hydroxide from Scharlau (Badalona, Spain), bacterial cell lysis buffer from NZYTech (Lisbon, Portugal), imidazole from Merck (Darmstadt, Germany), and fluorescein from Acros Organics (Geel, Belgium). QIAprep Spin Miniprep Kit and pQE-T7-2 vector were supplied by Qiagen (Hilden, Germany). Finally, HisTrap FF crude columns and PD-10 columns were purchased from Cytiva (Chicago, IL, USA).

Ultrapure water obtained from a Millipore Milli-*Q* water purification system was used throughout.

Stock solutions of the ISD at a 2 mg mL^–1^ concentration in DMSO stored at 4 °C were used to prepare standard solutions on a daily basis by dilution in PBST (10 mM, 0.05% T20, pH 7.4).

### Instrumentation

UV–vis absorption spectra were acquired using a Varian Cary 3-Bio spectrophotometer. Steady-state fluorescence measurements were made using a Horiba Fluoromax-4TCSPC spectrofluorometer equipped with a 150 W xenon lamp.

Emission lifetime measurements were made on an FLS980-Xd2-T spectrometer from Edinburgh Instruments (Livingston, UK) equipped with a Horiba 470LH diode laser (463 nm, < 1 ns pulse width) using a 460 nm excitation bandpass interference filter and a Hamamatsu R928P photomultiplier that was cooled thermoelectrically at −21 °C. Emission lifetime values as measured in the multi-channel scaling mode were extracted from the exponential decay data by using a nonlinear fitting algorithm in the software FAST (Edinburgh Instruments, v. 3.5.0). This allowed residuals with χ^2^ < 1.2 to be obtained. Emission measurements were made in optically diluted samples with *A*_max_ < 0.1 in an air atmosphere.

A CLARIOstar fluorescence reader from BMG Labtech (Ortenberg, Germany) was used for microplate measurements. The instrument was operated, and the data was processed by using MARS, the manufacturer’s original software. The excitation wavelength was 475 ± 10 nm, and detection was monitored at 520 ± 10 nm.

Solutions were centrifuged on a miniSpin microcentrifuge from Eppendorf AG (Hamburg, Germany) and evaporated on a DNA SpeedVac 110 apparatus from Savant Instruments (Holbrook,NY, USA), microplates were washed in a HydroFlex plate washer from Tecan (Männedorf, Switzerland) furnished with a magnetic support.

A QCM-Z500 quartz crystal microbalance (QCM) with impedance monitoring from KSV Instruments Ltd. (Helsinki, Finland) equipped with a thermoelectric module from Oven Instruments (Mechanicsburg, PA, USA) was used for the analysis of the FK506-protein interaction. The resonant frequency shift (Δ*f*) and change in energy dissipation (Δ*D*) of an Au-coated AT-cut 5 MHz sensor from Nordtest srl (Serravalle Scrivia, AL, Italy) were recorded at the resonance frequency (*f*_0_) and its 3rd, 5th, 7th, 9th, and 11th overtones. The active area of the sensor was 0.785 cm^2^. The temperature was kept constant at 20.0 ± 0.1 °C by using a Peltier element connected to the thermoelectric module. Changes in resonance frequency (Δ*f*) and energy dissipation (Δ*D*) were monitored through multiple odd overtones with a fundamental frequency of 5 MHz.

### Fusion Protein Characterization by NMR Spectroscopy

Saturation transfer difference (STD) curves were acquired from 2048 scans performed in D_2_O phosphate-buffered saline (PBS) buffer at 298 K on a Bruker 700 MHz spectrometer equipped with a cryoprobe. The sample concentration used was 0.3 mM for FK506 and 6 nM for the FKBP1A–EmGFP protein (ligand/protein ratio 50:1). STD at the resonance frequency was set to −0.3 ppm. Two-dimensional TOCSY and NOESY spectra were additionally acquired from the same samples for assignment purposes.

### Molecular Cloning

For the expression of FKBP1A fused with EmGFP, the encoding genes were PCR-amplified from the commercial plasmids by using the Phusion Hot Start II DNA polymerase and the primer sets stated in Table S1. The designed primer sets allowed the incorporation of a HisTag with a TEV site at the N-terminal of FKBP1A and also a GGGS linker between FKBP1A and EmGFP.

**Figure 2:**
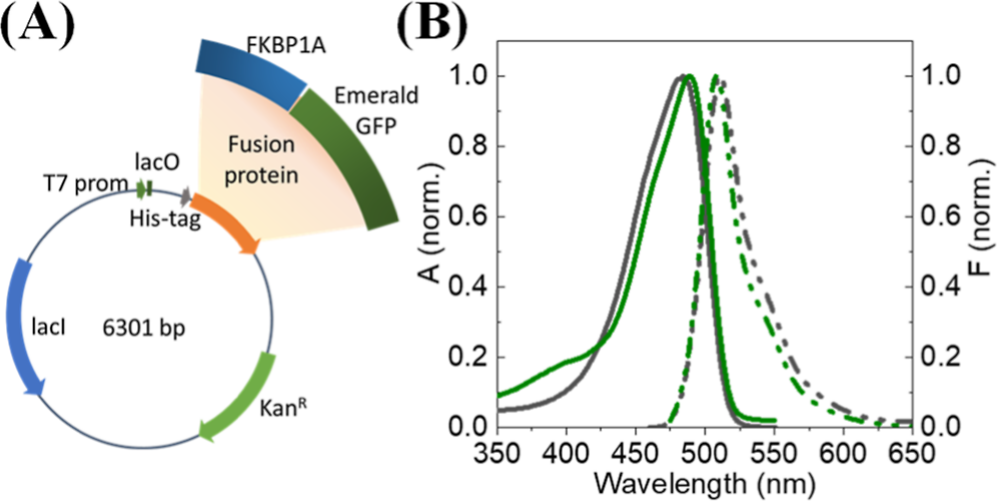
(A) Main features of the expression vector used to obtain a translational fusion protein consisting of FKBP1A and EmGFP. The vector pQE-T7-2 includes a T7 promotor, the *lac* repressor (*lacI*) and *lac* operator (*lacO*) to suppress uninduced expression, kanamycin resistance (*Kan*^*R*^), and a Histidine affinity tag (HisTag). (B) Normalized absorption and fluorescence spectra for EmGFP (green) and FKBP1A-EmGFP (gray) in PBS (10 mM, pH 7.4).

The amplified vector and gene DNA fragments were combined in a Gibson assembly reaction by using the NEBuilder HiFi DNA Assembly Master Mix according to the manufacturer’s instructions. The assembled plasmid (Figure 2A) was transformed into chemically competent DH5α *E. coli* cells by heat shocking and plated on LB-agar containing kanamycin 50 μg mL^–1^. A single colony was grown overnight in 5 mL of a LB-kanamycin solution of the same concentration, and plasmids were extracted and purified by using the QIAprep Spin Miniprep Kit. The coding sequence of the constructs was verified to ensure correct assembly by Sanger sequencing (Figure S2). The plasmid was transformed into chemically competent BL21 (DE3) *E. coli* cells by heat shock, and the resulting cells were plated and selected on LB-agar containing kanamycin 50 μg mL^–1^.

### Protein Expression and Purification

For FKBP1A–EmGFP expression, 5 mL of preculture (LB-kanamycin) was inoculated with a single colony harboring the plasmid and grown overnight at 37 °C. The preculture was used to inoculate a main culture of 200 mL (LB-kanamycin) to an optical density at 600 nm (OD_600_) of 0.05 and grown at 37 °C at 175 rpm until an OD_600_ value of 0.6–0.8 was reached. After the induction of FKBP1A–EmGFP-fused protein expression with 100 μL of IPTG (1 M), cultivation was allowed to continue at the same temperature for 16 h. Then, cells were collected by centrifugation at 5000*g* at 4 °C for 10 min and stored at −80 °C for at least 3 h before resuspension in NZY bacterial cell lysis buffer (approximately 5 mL g^–1^ cells) supplemented with DNaseI (4 μg mL^–1^), lysozyme (100 μg mL^–1^), and protease inhibitor cocktail and lysed by sonication on ice (VibraCell Ultrasonic Processor 130 W 20 kHz, Ampl 70%) using on/off pulse cycles of 10 s. Cell debris was removed by centrifugation at 15,000*g* at 4 °C for 15 min. The correct expression of the fluorescent protein was confirmed by examining the fluorescence emission in both soluble and insoluble fractions and culture media.

The recombinant fluorescent protein was purified from the cell lysate by the histidine tag (HisTag) present in the N-terminal using a HisTrap column according to the manufacturer’s instructions. Briefly, the lysate supernatant was diluted 1:3 (v/v) with binding buffer (20 mM phosphate, pH 7.4, containing 500 mM NaCl and 20 mM imidazole) and loaded onto the HisTrap column at 1 mL min^–1^. The column was washed with 30 mL of binding buffer prior to the elution of the protein with 20 mM phosphate buffer at pH 7.4 containing 500 mM NaCl and 500 mM imidazole. The presence of the fluorescent protein was monitored by measuring the fluorescence of 1 mL eluate fractions, and the fluorescent fractions were pooled.

Imidazole was removed by using a PD-10 column according to the manufacturer’s instructions, and the protein was stored in PBS buffer at 4 °C. Finally, protein purity was evaluated by sodium dodecyl sulfate–polyacrylamide gel electrophoresis analysis (Figure S3), and concentrations were calculated by using theoretical extinction coefficients at 280 nm (ExPASy ProtParam tool).

### Magnetic Bead Functionalization

FK506 was immobilized by using hydrophilic Dynabeads M-270 with amine groups (MB), and carboxylated FK506 (FK506-CO_2_H)^35^ was used in combination with EDC/sulfo-NHS chemistry. For this purpose, 2 mg of MB were washed three times with 500 μL of MES buffer (0.1 M, pH 4.7) and resuspended in 1 mL of MES buffer containing 0.8 μmol FK506-CO_2_H, 16.8 μmol EDC, and 35.02 μmol sulfo-NHS. After incubation for 18 h, MB were washed 3 times with 500 μL of MES and 2 times with 500 μL of PBST. Finally, FK506-functionalized MB were stored in 400 μL of PBST (5 mg mL^–1^) at 4 °C.

### Fluororeceptor-Based Bioassay

This bioassay was performed on FK506 standard solutions or spiked samples in the presence of FKBP–EmGFP and MB-FK506 at the optimal concentrations. Briefly, fluorescent fusion protein (20 μL, 52.5 μg mL^–1^) was mixed with 180 μL of either FK506 standard solution or extracted blood sample in a black 96-well plate and incubated for 25 min with slow agitation at 18 °C. Then, 10 μL of functionalized MB were added to the solution, and, after incubation for 15 min, MB present in the wells were washed three times with PBST in a HydroFlex plate washer and resuspended in 50 μL of buffer.

Fluorescence was monitored with a CLARIOstar microplate reader. Figure 1 depicts the workflow of the assay.

The fluorescence values obtained as the averages of 15 independent well measurements were normalized to the minimum and maximum signals for logarithmic plotting as a function of the FK506 concentration. Experimental data were fitted to the following four-parameter sigmoidal logistic equation by using the software Origin 2019
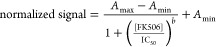
1

The limit of detection (LOD), which was taken to be the analyte concentration inhibiting the signal by 10%, and the dynamic range (DR) to encompass the analyte concentrations led to a normalized response over the range 20–80%.^36^

CR was calculated by substituting the optimum assay parameter values into the following equation
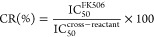
2where IC_50_ corresponds to 50% binding inhibition.

### Analysis of Human Blood Samples

Whole blood samples were obtained from organ transplant patients and healthy volunteers at the Clinical University Hospital of Valladolid, Spain. Samples were collected with permission of the hospital’s Ethics Committee (no. PI 21-2245) and kept in EDTA at −20 °C for transport and storage. FK506 was extracted by using a previously reported method with minor modifications.^37^ Briefly, 600 μL of sample was mixed with 600 μL of methanol, sonicated for 5 s three times, and centrifuged at 12,100*g* for 30 min. Then, the supernatant was evaporated to dryness and resuspended in 30 μL of PBS containing 1% T20 with bath sonication for 5 min. Reconstituted samples were centrifuged at 12,100*g* for 5 min, and the supernatant was diluted with PBS to a final volume of 600 μL. Treated samples were subjected to the bioluminescence assay and the results compared with those obtained by an external laboratory using the commercial ARCHITECT iSystem from Abbot.

## Results and Discussion

### Design and Characterization of Recombinant FKBP1A–EmGFP

EmGFP-tagged FKBP1A was obtained by cloning the FKBP1A gene in fusion with EmGFP using standard molecular biology techniques.^38^ EmGFP and the FKBP1A receptor in the immunophilin protein EmGFP construct (Figure 2A) were separated with a glycine–serine linker (GGGS) to facilitate the FK506 binding. Also, a HisTag was included at the N-terminus to facilitate protein purification by affinity chromatography.

PCR-amplified genes were successfully fused in the pQE-T7-2 vector by using the Gibson assembly reaction, and FKBP1A–EmGFP was overexpressed as a soluble form in *E. coli* BL21 (DE3).

Figure 2B shows the absorption and emission spectra for FKBP1A–EmGFP in saline phosphate buffer (PBS, 10 mM, pH 7.4). The absorption and fluorescence peak fell at 484 and 511 nm, respectively. The fluorescence quantum yield, determined against fluorescein as the standard (Φ_f_ = 0.89 ± 0.04 in NaOH),^39^ was 0.70 ± 0.02 in PBS and the excitation wavelength λ_exc_ = 450 nm. All measurements were made in triplicate, and absorption at the excitation wavelength was always below 0.1. The fluorescence lifetime in PBS was 3.07 ± 0.03 ns (Figure S4).

The results for FKBP1A–EmGFP are consistent with the reported data for EmGFP, which exhibits an absorption peak at 487 nm, an emission peak at 509 nm, and a fluorescent quantum yield of 0.68 in aqueous solutions.^34^ The fact that the shape and position of the fluorescence peaks remained unchanged indicates that fusion with the FKBP1A protein did not alter the structure of EmGFP.

### Analysis of the FK506–Protein Interaction by QCM

**Figure 3:**
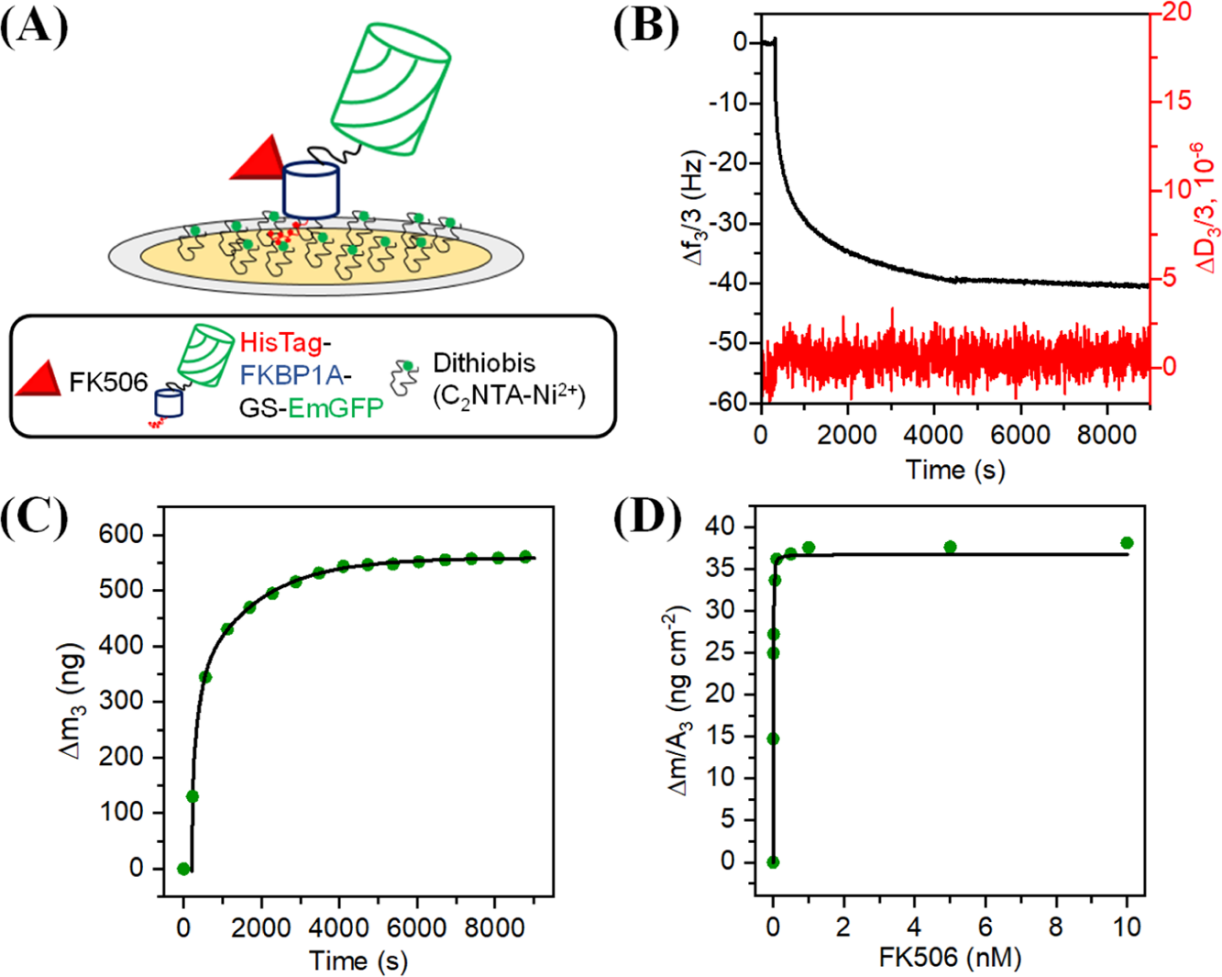
(A) Sensor nanoplatform used to examine FK506 binding to FKBP1A through the HisTag-FKBP1A-EmGFP fusion protein. (B) Normalized frequency shift (black curve) and variation of energy dissipation (red curve) during the adsorption of a 500 nM solution of the HisTag-FKBP1A-EmGFP protein on the dithiobis(C_2_NTA-Ni^2+^) layer. (C) Variation of adsorbed protein mass on dithiobis(C_2_NTA)-Ni SAM with time. (D) Change in surface mass density as a function of FK506 concentration, with the solid line representing the Langmuir fitting curve.

Binding of FK506 to the recombinant protein FKBP1A–EmGFP was examined by using a QCM equipped with a sensor chip that was functionalized with a nanostructured layer of dithio*bis*(C_2_NTA-Ni^2+^) to provide an immobilization platform for the fusion protein by HisTag (Figure 3A–C; see Supporting Information for a detailed description of the results).

Once the protein layer formed, increasing concentrations of FK506 from 1 pM to 10 nM were added to the QCM measuring chamber until a constant frequency shift was observed. Figure 3D shows variation of the mass surface density resulting from the frequency shift (Δ*f*_3_/3) as a function of the FK506 concentration. As can be seen, the analyte was firmly captured by the sensor surface. FK506 seemingly induced no changes in viscoelastic properties in the protein surface layers. Kinetic parameters were calculated in accordance with the Langmuir binding model.

The reported solution dissociation constants for FKP1A binding to FK506^40^ and Sir^41^ range from 0.6 to 0.9 nM; however, the measured FKBP1A–EmGFP binding constant was extremely small (*K*_d_ = 2.1 ± 0.3 pM) as a result of the protein being immobilized with a restricted conformation—and of the tests not being comparable with determinations in solution.

### Analysis of the FK506 Interaction with FKBP1A–EmGFP by NMR Spectroscopy

**Figure 4:**
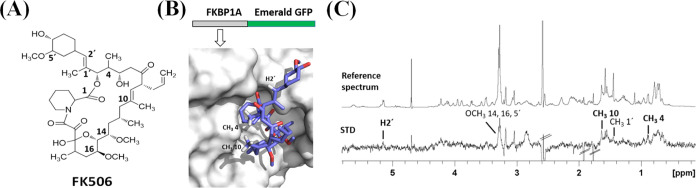
(A) Chemical structure of FK506. (B) X-ray structure of the FKBP1A–FK506 complex (PDB code 5HUA). (C) STD and reference NMR spectra for a sample containing FKBP1A–EmGFP fusion protein and FK506. The strongest STD signals are highlighted in bold text. Solvent residual signals, labeled with parallel lines, appeared as cancelled STD signals.

Characterizing the interaction of FK506 with FKBP1A–EmGFP by nuclear magnetic resonance (NMR) spectroscopy involved acquiring STD signals. This experiment allows detecting the signals of the hydrogens that are interacting with the protein; the signals of the non-interacting hydrogens are cancelled out. Based on the results (Figure 4), FK506 was effectively recognized by the FKBP1A–EmGFP fusion protein. Also, the results were consistent with the structure of the FKBP1A–FK506 complex as determined by X-ray crystallography (PDB 5HUA);^42^ in fact, the strongest STD signals (viz., H2′, CH_3_ 4, and CH_3_ 10) were due to the ligand protons pointing toward the protein surface in the structure (Figure 4B).

### Assay Optimization

Developing a simple, fast method for determining FK506 required optimizing the operating conditions of the bioassay. Different combinations of the amounts of MB-FK506 (2–8 μg/well) and fluorescent protein (1.3–6.7 μg mL^–1^) were used to maximize the sensitivity while ensuring good precision and low nonspecific binding. As can be seen from Figure S7, the best results were obtained with 7 μg of MB-FK506 per well and an FKBP1A–EmGFP concentration of 5.25 μg mL^–1^. This combination afforded good sensitivity and a wide DR.

Recognition of FK506 by the recombinant protein was found to depend strongly on the assay conditions, which affected the interactions involved. The optimal buffer composition was identified by using the previous conditions in the absence (*B*_0_) and presence of a 50 ng mL^–1^ concentration of FK506 (*B*_50_). As can be seen from Figure S8, PBS, HEPES, and TRIS resulted in a highly nonspecific surface binding of the FKBP1A–EmGFP protein to the magnetic beads. However, adding 0.05% Tween 20 (T20) to the PBS buffer (PBST) minimized nonselective hydrophobic interactions between MB-FK506 and the fusion protein. PBST was thus selected for further testing as it provided the best results in terms of sensitivity (lowest *B*/*B*_0_ ratio) and DR.

The influence of the incubation time on the analytical response was examined by incubating the FK506 concentrations over the range 0–500 ng mL^–1^ with FKBP1A–EmGFP for 15, 25, or 35 min before MB-FK506 was added. As shown in Figure S9, increasing the incubation time from 15 to 25 min increased the sensitivity. Longer times, however, provided no additional advantage, so a time of 25 min was chosen for further testing. MB-FK506 stability was assessed by monitoring a batch over consecutive days. Functionalized particles remained stable for at least 6 days provided that they were stored at 4 °C.

### Analytical Characterization

**Figure 5:**
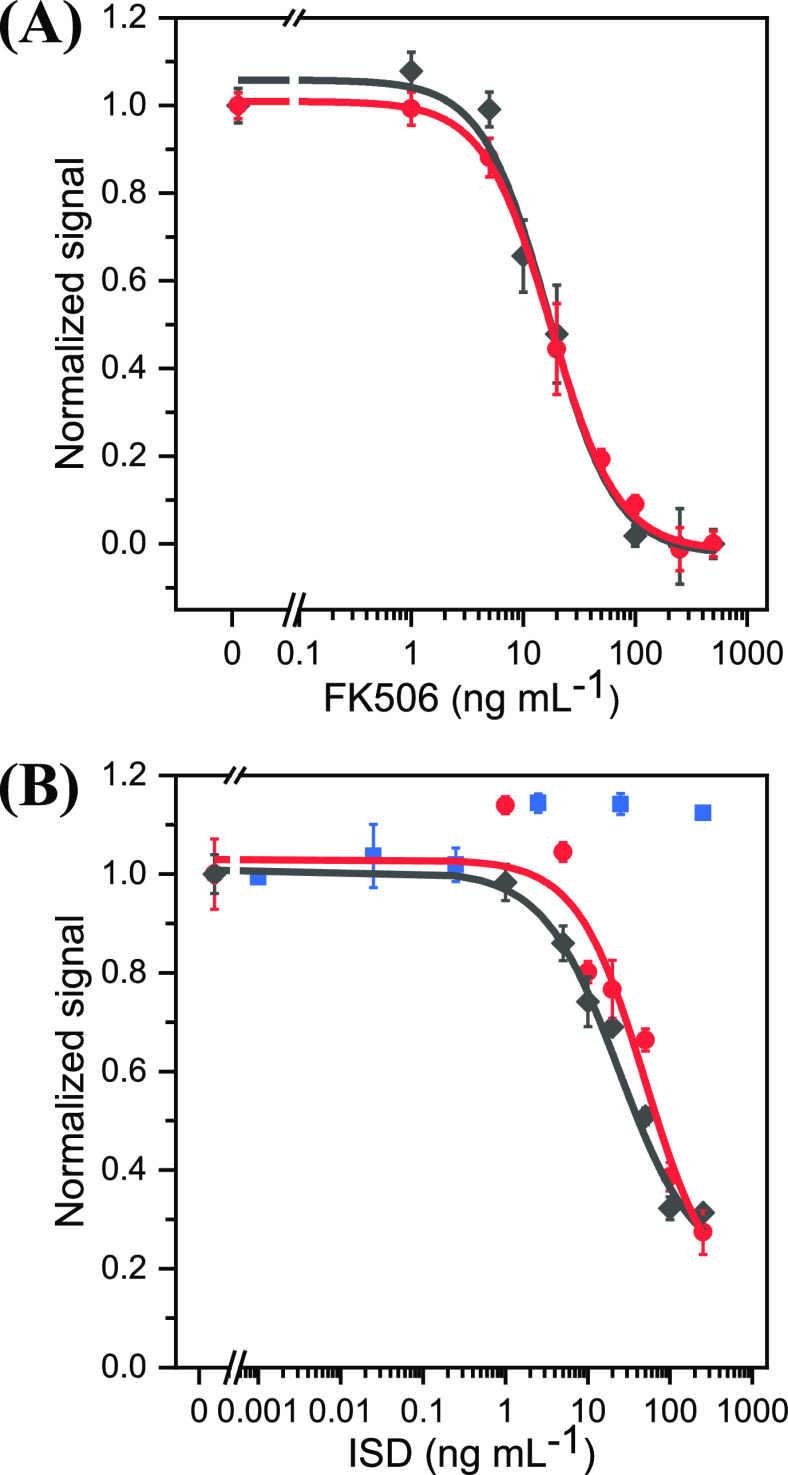
(A) Calibration plots for FK506 in phosphate buffer (10 mM, pH 7.4) supplied with 0.05% T20 (black ◆, *n* = 3) and in extracted whole blood (red ●, *n* = 3), as obtained by using the recombinant protein FKBP1A–EmGFP and MB-FK506 in PBST. (B) Dose–response plots for FK506 (red ●, *n* = 3, 8 points), MPA (blue ■, *n* = 3, 7 points), and Sir (black ◆, *n* = 3, 8 points) obtained by using FKBP1A–EmGFP as the recognizing element and MB-FK506 in PBST. The results are mean signals ± standard errors of the mean (*n* = 3).

Figure 5A shows the normalized competition curve obtained with FK506 standards at concentrations from 0 to 500 ng mL^–1^ in PBST. IC_50_ was 19 ng mL^–1^ and the LOD, as calculated at 10% inhibition, 3 ng mL^–1^. The DR, taken as 20–80% inhibition,^36^ spanned concentrations from 5 to 70 ng mL^–1^. The average within-day relative standard deviation (RSD, *n* = 3) was 11% and the between-day RSD for assays performed on four nonconsecutive days < 12%. As can be seen from Table S5, the proposed method is slightly less sensitive than some commercial immunoassays, whose LOQ range from 0.08 to 0.7 ng mL^–1^. However, it has a wider DR (Table S5), and its measuring protocol can be easily automated. Moreover, the LOD meets the medical specifications for detection and quantification of the drug in blood; the recommended therapeutic dose for patients with a normal rejection risk usually ranges from 5 to 20 ng mL^–1^.^3^

Assay selectivity was assessed by determining two ISDs typically co-administered with FK506 in transplanted patients, viz., MPA and Sir (Figure S1). As can be seen from Figure 5B, MPA exhibited negligible CR (<1%). On the other hand, Sir induced a response similar to that of FK506 (IC_50_ = 50 ng mL^–1^; CR = 38%). Sir, which is structurally similar to FK506, is also recognized by the receptor in the human body. Thus, it forms a complex that inhibits the mammalian target of rapamycin, thereby altering the proliferation of T lymphocytes and diminishing antibody production as a result.^43,44^ In any case, the assay allows either drug to be quantified in transplant patients.

### Evaluation of the Matrix Effect

Matrix effects were assessed in blood samples subjected to the extraction procedure described above and spiked with increasing concentrations of FK506 from 0 to 500 ng mL^–1^ before the analysis. Figure 5A compares the dose–response curves obtained in buffer and pretreated blood. No significant differences (*p* > 0.05) were observed, so no dilution was required to analyze the real samples.

### Analysis of Samples

The efficiency of the extraction procedure was assessed by using the optimized fluororeceptor-based bioassay to analyze blank whole blood samples spiked at four different concentration levels (10, 13, 16, and 20 ng mL^–1^). Samples were extracted with MeOH under ultrasound, evaporated, and reconstituted in PBST prior to the analysis. As can be seen from Table S4, there were no significant differences between measured FK506 concentrations and those of immunosuppressant added to the blank samples at any concentration level.

**Figure 6:**
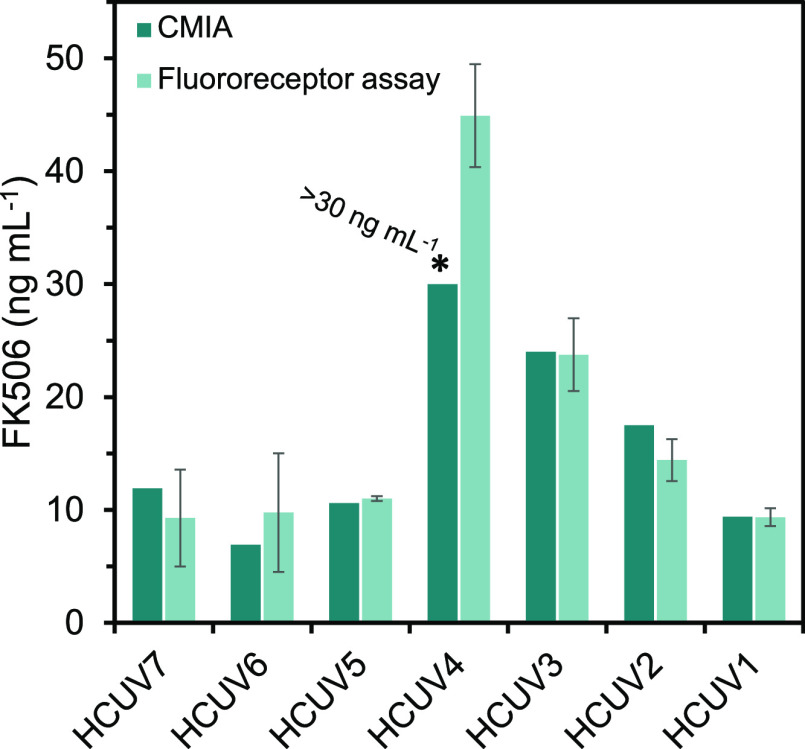
Comparison of the performance of the proposed fluororeceptor-based assay and the commercial CMIA (RSD ≤ 10%) for quantification of FK506 in whole blood samples from transplant patients.

Finally, the suitability of the proposed bioassay for FK506 quantification in whole blood from transplant patients (Table S6) was evaluated, and the results were compared with those obtained by an external laboratory using the commercial ARCHITECT immunoassay System from Abbott. As can be seen from Figure 6, there were no significant differences between the results obtained with the optimized method and the commercial assay, which testifies to the suitability of the proposed bioassay. Sample HCUV4 contained an analyte concentration exceeding 30 ng/mL, so it could not be quantified with the commercial system. By contrast, the proposed fluororeceptor-based bioassay allowed it to be quantified, thanks to its wide DR. No FK506 was detected in the control samples.

## Conclusions

As shown here, recombinant FKBP1A–EmGFP provides an effective alternative to antibodies for detection and quantification of FK506 in human whole blood samples. Recombinant fusion between FKBP1A and EmGFP avoids the need for chemical conjugation, thus providing an unlimited production system that avoids batch-to-batch variability. No matrix effect was observed in samples treated with methanol, evaporated, and reconstituted in PBST. Also, up to 96 treated samples can be analyzed within 45 min by using a microplate reader. The optimized fluororeceptor-based assay provides good sensitivity and was successfully used to analyze the samples from organ transplant patients treated with FK506. The results compared favorably with those of a commercial assay typically used by clinical laboratories to monitor the FK506 concentration.

## Supporting Information Available

Chemical structures of the ISDs used in the assays; details of the primers sets used for PCR amplification; Sanger sequencing aligment and SDS-PAGE analysis of FKBP–EmGFP protein, fluorescence decay profile of the recombinant protein; MALFI-TOF analysis of FKBP1A–EmGFP; details about the preparation of the FK506 protein platform on the QCM sensor and data analysis; optimization of the concentration of the fluorescent protein and functionalized magnetic beads; effect of the buffer solution and the incubation time; comparative summary of analytical methods reported for the analysis of FK506 in biological samples; and method validation in whole blood samples (PDF)
