# Supplementary material for: Biosensing Tacrolimus in Human Whole Blood by Using a Drug Receptor Fused to the Emerald Green Fluorescent Protein
Source: Anal Chem. 2022 Nov 16;94(47):16337–44. doi: 10.1021/acs.analchem.2c03122 (PMC9716524; doi:10.1021/acs.analchem.2c03122)
Supplement: Supplementary file 1 — ac2c03122_si_001.pdf [file ac2c03122_si_001.pdf]

Supporting Information

Biosensing Tacrolimus in Human Whole Blood by Using a Drug Receptor Fused to the Emerald Green Fluorescent Protein

Bettina Glahn-Martínez,[†] Giacomo Lucchesi,[‡] Fernando Pradanas-González,[†] Ana Isabel Manzano,[⊥] Ángeles Canales,[⊥] Gabriella Caminati,[‡] Elena Benito-Peña,[†]* María C. Moreno-Bondi[†]*

[†] *Department of Analytical Chemistry, Faculty of Chemistry, Universidad Complutense de Madrid, Plaza de las Ciencias, Ciudad Universitaria, 28040 Madrid, Spain*

[‡] *Department of Chemistry "Ugo Schiff" and CSGI, University of Florence, Via della Lastruccia 13, 50019 Sesto Fiorentino, Italy*

[⊥] *Department of Organic Chemistry, Faculty of Chemistry, Universidad Complutense de Madrid, Plaza de las Ciencias, Ciudad Universitaria, 28040 Madrid, Spain*

Contents

Figure S1 ..... **S2**
Table S1 ..... **S2**
Figure S2 ..... **S2**
Figure S3 ..... **S3**
Figure S4 ..... **S3**
Protein identification by MALDI-TOF mass spectrometry **S4**
  Table S2 ..... **S4**
  Figure S5 ..... **S5**
Preparation of the FK506-protein platform on the QCM sensor **S6**
  Figure S6 ..... **S8**
  Table S3 ..... **S8**
Figure S7 ..... **S10**
Figure S8 ..... **S10**
Figure S9 ..... **S11**
Table S4 ..... **S11**
Table S5 ..... **S12**
Table S6 ..... **S14**

References **S15**

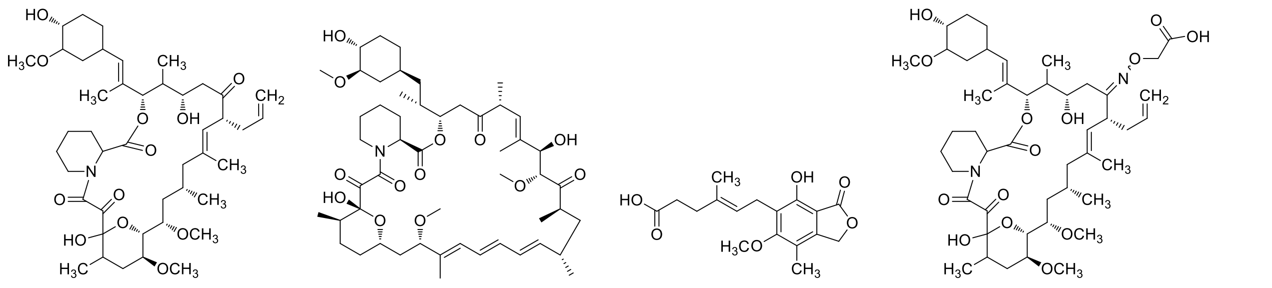

FK506 **Sir** **MPA** **FK506-CO_2H**

Figure S1. Chemical structures of the immunosuppressant drugs tacrolimus (FK506), mycophenolic acid (MPA) and sirolimus (Sir), and the carboxylated derivative of tacrolimus (FK506-CO_2H).

Table S1. Primer sets used for PCR amplification of the pQE-T7-2 vector and gene fragments. The oligo tail added to the target gene sequence is in bold face, the segment identical with the target gene in italics and the overlapping regions for the Gibson assembly reaction underlined.

Vector	Primer	Sequence 5′->3′
FKBP1A plasmid	FP	**ATC ACC ATG AAA ACC TGT ATT TTC AGT CC***G GAG TGC AGG TG*
	RP	**TTG CTC ACC GAA CCT CCA CC***T TCC AGT TTT AGA AG*
EmGFP plasmid	FP	**GGT GGA GGT TCG** *GTG AGC AAG GGC GAG*
	RP	**GCA GCC GGA TCT TAC** *TAC TTG TAC AGC TCG TC*
pQE-T7-2 backbone	FP	*TAG TAA GAT CCG GCT G*
	RP	**ATA CAG GTT TTC ATG GTG ATG GTG ATG ATG** *CAT ATG ATT TAT ATC TCC TTC TT*

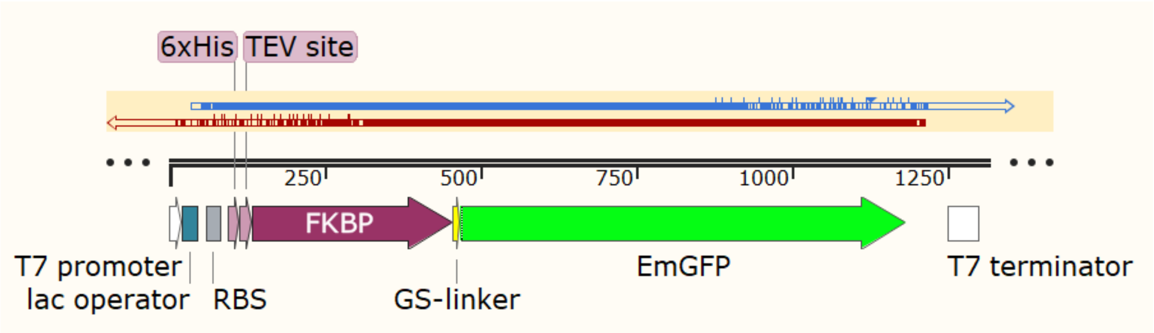

Figure S2. Alignment of the gene sequence of the recombinant FKBP-EmGFP protein (bottom scheme) with the sequences obtained by the Sanger sequencing with the direct (blue line) and reverse (red line) primers. The colored parts of the upper arrows indicate the homologous areas of the sequences. Sanger sequencing was carried out at the Genomics and Proteomics Research Support Center of Complutense University and the alignment was done with the SnapGene 3.2.1 software (GSL Biotech).

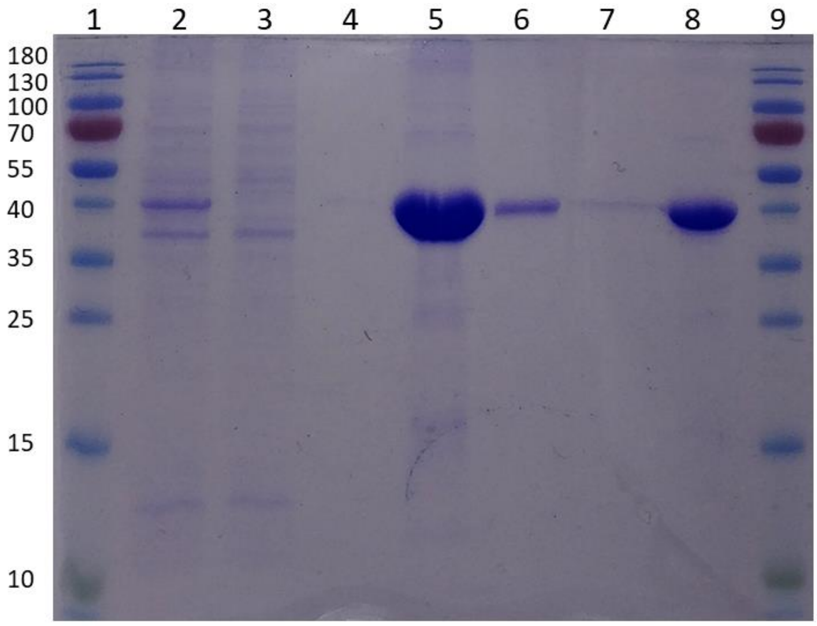

Figure S3. SDS-PAGE analysis of the purified fusion protein with Coomassie brilliant blue protein staining: lane 1 and 9, molecular marker (Thermo Scientific™ PageRuler™ Prestained Protein Ladder, 10 to 180 kDa); 2, lysate sample; 3, HisTrap column throughput with non-retained proteins; 4-7, aliquots from the HisTrap column elution; 8, purified protein obtained after PD-10 column. Prior to analysis, a 5-minute boiling step at +95 °C have been used. The thickest band corresponds to the molecular weight of the fusion protein (~40.6 kDa).

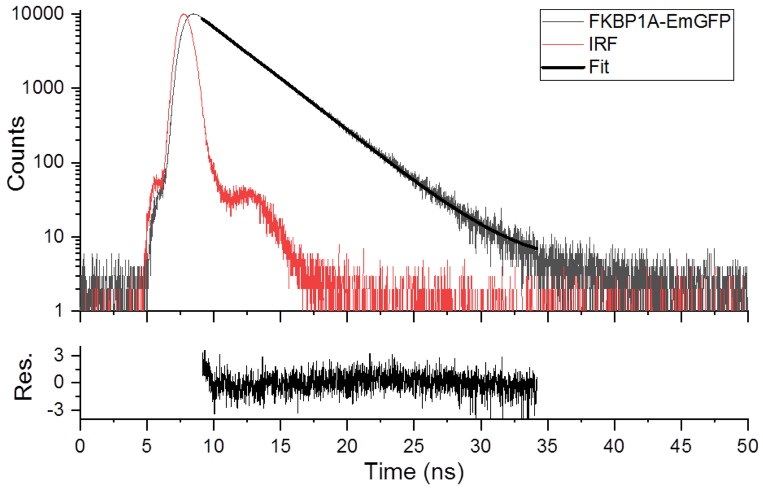

Figure S4. Fluorescence decay profile of the FKBP1A-EmGFP protein in PBS at 25 ºC. IRF: Instrument Response Function.

Protein identification by MALDI-TOF mass spectrometry

MALDI-TOF MS analysis of the electrophoretically resolved protein was performed, at the Proteomics Unit of the Complutensian University of Madrid, on a 4800 Plus Proteomics Analyzer MALDI-TOF/TOF mass spectrometer (Applied Biosystems, MDS Sciex, Toronto, Canada). The protein was excised from the SDS-PAGE gel, in-gel reduced, alkylated and digested with trypsin. The MALDI-TOF/TOF MS technique provides peptide mass fingerprints (**Figure S5A**), those peptides were collated and represented as a list of monoisotopic molecular weights and compared with the masses of theoretical trypsin digestion of the recombinant protein (**Figure S5B and Table S2**). The results showed a coverage of 63% of the protein, having the FKBP1A domain a coverage of more than 87% of the primary sequence. Furthermore, the EmGFP domain was also observed except the C-terminal end that contains low number of Lys and Arg avoiding the digestion by Trypsin that produces too large peptides to be identified by MS.

Table S2. Information of peptides identified form the recombinant FKBP1A-EmGFP after a trypsin digestion and MALDI-TOF MS analysis.

Start - End	Observed	Mr(expt)	Mr(calc)	ppm	M'	Peptide
1-27	3149.4546	3148.4473	3148.4435	1.23	0	-.MHHHHHHENLYFQSGVQVETISPGDGR.T
1-27	3165.4644	3164.4571	3164.4384	5.91	0	-.MHHHHHHENLYFQSGVQVETISPGDGR.T + Oxidation (M)
32-48	1950.9281	1949.9208	1949.9088	6.18	1	K.RGQTCVVHYTGMLEDGK.K
32-48	1966.9147	1965.9074	1965.9037	1.88	1	K.RGQTCVVHYTGMLEDGK.K + Oxidation (M)
33-48	1794.8073	1793.8000	1793.8077	-4.29	0	R.GQTCVVHYTGMLEDGK.K
33-49	1922.8994	1921.8921	1921.9026	-5.46	1	R.GQTCVVHYTGMLEDGKK.F
55-61	904.4854	903.4781	903.4926	-16.1	1	R.DRNKPFK.F
62-71	1220.6844	1219.6772	1219.6747	2.03	1	K.FMLGKQEVIR.G
67-85	2159.0652	2158.0579	2158.0589	-0.47	1	K.QEVIRGWEEGVAQMSVGQR.A
72-85	1533.7186	1532.7114	1532.7042	4.68	0	R.GWEEGVAQMSVGQR.A
72-85	1549.7126	1548.7054	1548.6991	4.05	0	R.GWEEGVAQMSVGQR.A + Oxidation (M)
88-119	3405.7754	3404.7681	3404.8020	-9.96	0	K.LTISPDYAYGATGHPGIIPPHATLVFDVELLK.L
129-151	2437.2913	2436.2840	2436.2537	12.4	0	K.GEELFTGVVPILVELDGDVNGHK.F
152-166	1503.6492	1502.6419	1502.6525	-7.08	0	K.FSVSGEGEGDATYGK.L
152-170	1958.9813	1957.9740	1957.9633	5.50	1	K.FSVSGEGEGDATYGKLTLK.F
199-210	1608.7136	1607.7063	1607.7191	-7.93	1	R.YPDHMKQHDFFK.S + Oxidation (M)
211-221	1266.5757	1265.5684	1265.5710	-2.06	0	K.SAMPEGYVQER.T
211-221	1282.5677	1281.5605	1281.5659	-4.26	0	K.SAMPEGYVQER.T + Oxidation (M)
222-232	1347.6522	1346.6449	1346.6507	-4.25	1	R.TIFFKDDGNYK.T
235-247	1477.7542	1476.7469	1476.7572	-7.02	1	R.AEVKFEGDTLVNR.I
239-247	1050.5089	1049.5016	1049.5142	-11.9	0	K.FEGDTLVNR.I
252-265	1542.7926	1541.7853	1541.7838	0.99	1	K.GIDFKEDGNILGHK.L

Table S2 (continued).

257-274	2130.9939	2129.9866	2130.0130	-12.4	1	K.EDGNILGHKLEYNYNSHK.V
266-274	1167.5317	1166.5245	1166.5356	-9.55	0	K.LEYNYNSHK.V
275-283	1065.5996	1064.5923	1064.5866	5.39	1	K.VYITADKQK.N

M‘: missed cleveage sites.

(A)

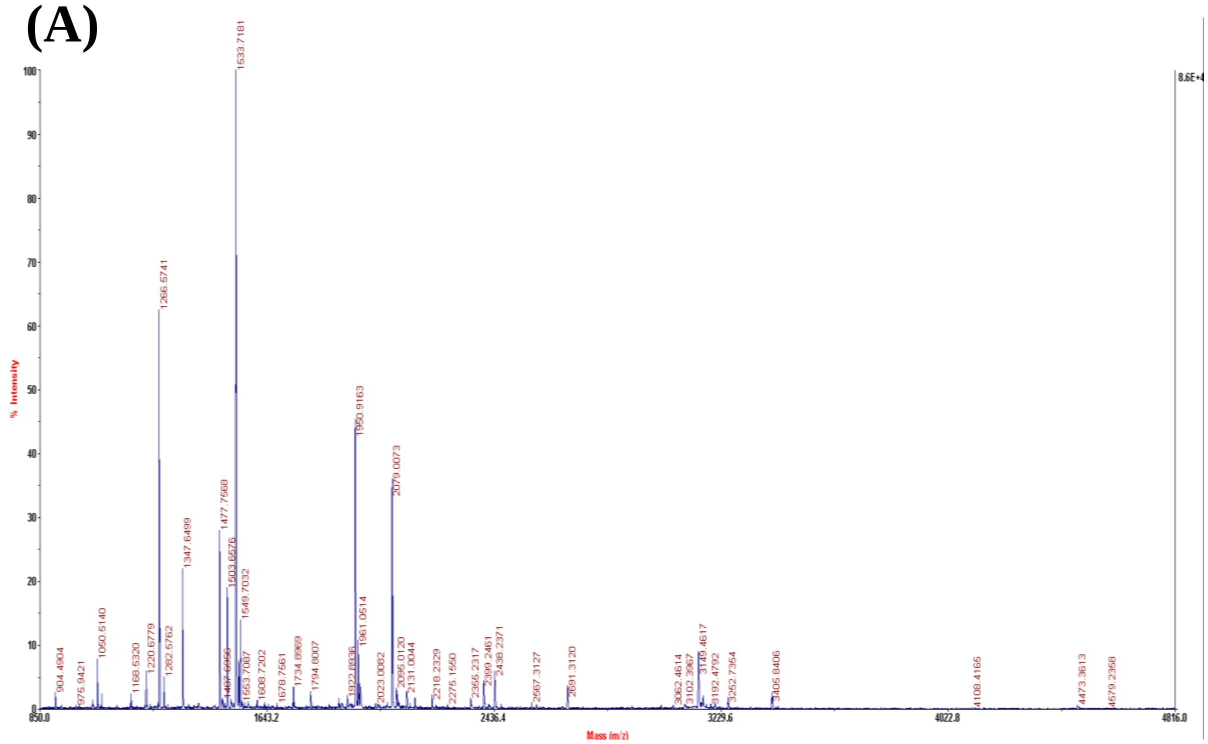

(B)

1 MHHHHHHENL YFQSGVQVET ISPGDGRTFP KRGQTCVVHY TGMLEDGKKF

51 DSSRDRNKPF KFMLGKQEVI RGWEEGVAQM SVGQRAKLTI SPDYAYGATG

101 HPGIIPPHAT LVFDVELLKL EGGGSVSKGE ELFTGVVPIL VELDGDVNGH

151 KFSVSGEGEG DATYGKLTLK FICTTGKLPV PWPTLVTTLT YGVQCFARYP

201 DHMKQHDFFK SAMPEGYVQE RTIFFKDDGN YKTRAEVKFE GDTLVNRIEL

251 KGIDFKEDGN ILGHKLEYNY NSHKVYITAD KQKNGIKVNF KTRHNIEDGS

301 VQLADHYQQN TPIGDGPVLL PDNHYLSTQS ALSKDPNEKR DHMVLLEFVT

351 AAGITLGMDE LYK

Figure S5. (A) MALDI-TOF mass spectrum of the FKBP1A-EmGFP eluted protein. **(B)** Sequence coverage of matched peptides (bold red) in FKBP1A-EmGFP. Marked amino acids correspond to the theorical cleavage sides of trypsin enzyme (C-term side of K and R).

Preparation of the FK506-protein platform on the QCM sensor and data analysis

QCM data analysis. The sensed mass of adsorbed protein layers was estimated using the Sauerbrey equation [1] (Eq. S1) and the Voigt model [2] supplied by QCMBrowser 2.30 software (KSV Instrument Ltd, Helsinki, Finland).

$$\Delta f = -\frac{2nf_0^2}{A\sqrt{\rho_q \mu_q}}\Delta m \qquad \text{Eq. S1}$$

where *n* is the overtone number and ρ_q and μ_q are the density and the shear modulus of the quartz crystal, respectively.

While the Sauerbrey equation was used for calculating the sensed mass of the rigid film with small or no energy dissipation, the Voigt model was applied in the case of larger dissipation values corresponding to soft and elastic layers.[3]

The surface density of the sample at saturation, and the relative molecular area occupied on the surface, $\bar{A}$, was obtained from Eq. S2

$$\bar{A} = \frac{MW}{\left(\frac{\Delta m}{A}\right)_{Sat} \cdot N_A} \qquad \text{Eq. S2}$$

where *MW* is the molecular weight of the adsorbed molecule, $(\Delta m/A)_{Sat}$ is the surface mass density at monolayer saturation and N_A is the Avogadro number.

Thickness of the adsorbed layer was determined from the detailed analysis of all overtones for the QCM-Z set-up using the QCMBrowse software and by simple estimation of the layer thickness using an average density of the adsorbed proteins obtained from QCMBrowse data fitting.

The data were analyzed with Igor 6.10A software (Wavemetrics, Lake Oswego, OR, USA).

The calculation of the kinetic parameters was done with a Langmuir binding model (Eq. S3):

$$\frac{\Delta m}{A} = \frac{\left(\frac{\Delta m}{A}\right)_{Sat}[FK506]}{k'_d + [FK506]} \qquad \text{Eq. S3}$$

where $(\Delta m/A)_{Sat}$ is the saturation surface mass density, k'_d is the pseudo binding constant for FK506 binding to FKP1A containing proteins.

Formation of dithiobis(C_2NTA-Ni^{2+}) nanoplatforms. Self Assembled Monolayer (SAM) of dithiobis(C_2NTA)-Ni^{2+} complex was formed on the QCM sensors using a procedure previously described.[4] Briefly, Au coated AT-cut 5 MHz sensor (Nordtest.srl, Serravalle Scrivia (AL), Italy) were cleaned with a drop of chromic acid solution for a few seconds, rinsed copiously with water and dried with a nitrogen flux. Then, the SAM was obtained with a two-step procedure: the chemisorption of dithiobis(C_2NTA) covalently immobilized on the sensor surface via sulfur-gold binding, followed by the addition of a Ni_2SO_4 solution resulting in complexation of Ni^{2+} to the water-exposed NTA groups.

The average orientation of the dithiobis(C_2NTA-Ni^{2+}) covalently immobilized on the sensor surface is reported in **Figure S6**. The procedure provides densely packed and rigid SAM of dithiobis(C_2NTA-Ni^{2+}) layers on gold-coated QCM sensors, in such nanoplatform Ni-NTA functional groups protrude exposing the two chelating aquoions available for further complexation.

The principal parameters obtained from the adsorption (summarized in **Table S3**) are in agreement with previous results on the same system which reported a surface mass density of about 95 ng/cm^2 and a rigid film thickness of about 16 Å.[4,5]

Immobilization of FKBP1A-containing protein. Solution of the fusion proteins (500 nM) in phosphate buffer were added to the QCM measuring chamber containing the dithiobis(C_2NTA-Ni^{2+}) modified QCM sensor. Consecutive additions were performed until a constant resonance frequency was reached; the chamber was then rinsed with phosphate buffer to remove protein excess or loosely bound protein.

Figure 3B represents the trend of the resonant frequency shift and the variation in energy dissipation of the third harmonic. The decrease in the frequency shift correlates with an increase of adsorbed mass on the sensor indicating that the protein adsorbs on dithiobis(C_2NTA-Ni^{2+}) layer likely interacting via histidine-tag complexation with the free coordination sites of the surface-immobilized Ni^{2+} ions.[6] The slight increase of ΔD_3 ($0.5\cdot10^{-6}$) shows that adsorption of the protein does not significantly affect the viscoelastic properties of the SAM layer. This finding suggests that a compact protein layer is formed on the surface.

As shown in **Figure 3C**, the adsorption of the FKBP1A-EmGFP takes around 2.5 h to be completed but the kinetics does not follow a simple mono-exponential dependence on time as expected for Langmuir adsorption. The presence of different kinetic regimes for protein adsorption was observed also by other authors.[7] In the first regime, the protein is arranged in a flat conformation occupying a large surface area, as adsorption proceeds the flip-up from the flat-lying conformation to a tilted one is promoted, further His-tagged proteins bind to the platform saturating the binding sites. However, the application of this model to our systems was not completely satisfactory, and the best agreement was found using the tri-exponential function (Eq. S4)

$$\Delta m = y_0 + A_1 e^{-\frac{x-x_0}{\tau_1}} + A_2 e^{-\frac{x-x_0}{\tau_2}} + A_3 e^{-\frac{x-x_0}{\tau_3}} \qquad \text{Eq. S4}$$

where y_0 is the saturation value, x_0 is the x offset, A_1, A_2 and A_3 are the amplitude and τ_1, τ_2 and τ_3 are the time constants expressed in seconds.

The reported data show that the first two-steps for protein adsorption usually proposed in the literature, namely chemisorption in flat-lying configuration (τ_1 = 13.569 s) and tilting up (τ_2 = 168.71 s) of the molecule for dense monolayer packing, is followed by a slower reorganization of the two protein subunits in the surface layer (τ_3 = 1539 s) to reach the maximum packing density.

The adsorbed mass of the protein, the area occupied by each molecule at surface saturation, the thickness increment caused by the adsorption of the protein and the change in dissipation obtained from the experimental data are reported in **Table S3**.

The average molecular area for the protein is in accordance with theoretical calculations reported in literature for similar systems containing EmGFP histidine tagged protein, where the average area ranges between 500 and 1250 Å^2/molecule depending on the orientation of the protein on the surface.[8]

Further insight in the protein conformation at the interface was obtained from the comparative analysis of the structural experimental data (thickness and molecular area at saturation) with the physical dimensions estimated from the chemical structure of the proteins. Given that the kinetic measurements proved that the proteins do not lay flat at the interface at maximum coverage, such conformations were excluded in the computations. Under this assumption, comparison of the experimental value of **Table S3** and the theoretical calculation allows us to draw some general conclusions. **Figure S6C** reports the most likely conformation of the system, compatible with the multistep kinetic. The FKBP1A protein is vertically oriented while the long axis of EmGFP is oriented with a tilt angle of 10° with respect to the normal to the interface.

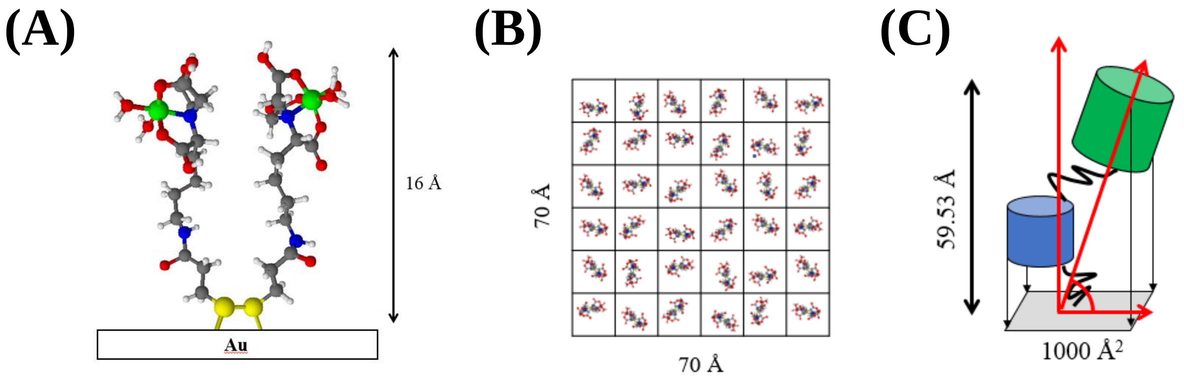

Figure S6. (A) Chemical structure of the dithiobis(C_2NTA-Ni^{2+}); **(B)** schematic representation of the surface density of dithiobis(C_2NTA-Ni^{2+}) on the Au-QCM sensor, **(C)** typical representation of the fusion protein conformation at the interface. The 3D structure of dithiobis(C_2NTA-Ni^{2+}) was obtained by means of the free software Chemsketch (ACD/Labs).

Table S3. Principal parameters obtained from the adsorption of dithiobis(C_2NTA) for the studied system and the FKBP1A modified protein on the C2NTA-Ni^{2+} substrate.

	Dithiobis(C_2NTA-Ni^{2+})	HisTag-FKBP1A-EmGFP
Surface mass density (ng/cm^2)	139 ± 20	677
Adsorbed mass (ng)	109 ±16	531
Average molecular area (Å^2)	90± 22	997
$\Delta D_3/3\cdot10^{-6}$	0.2 ± 0.3	1.0
Thickness increment, Å	16 ± 1	66
Ratio	1.15 ± 0.31	10

Binding of FK506 to the FKBP1A-containing protein. Sequential aliquots of tacrolimus solution at different concentrations ranging between $1 \cdot 10^{-12}$ M and $1 \cdot 10^{-8}$ M in phosphate buffer (10 mM, pH 7.4) were added to the QCM measuring chamber using the protein-functionalized QCM sensor (**Figure 3A**). Δf and ΔD were recorded as a function of time until constant values were reached for all the examined concentrations. The adsorbed mass after buffer rinsing was obtained from the normalized third overtone of the frequency shift ($\Delta f_3/3$) applying Sauberbrey equation (Eq. S1).

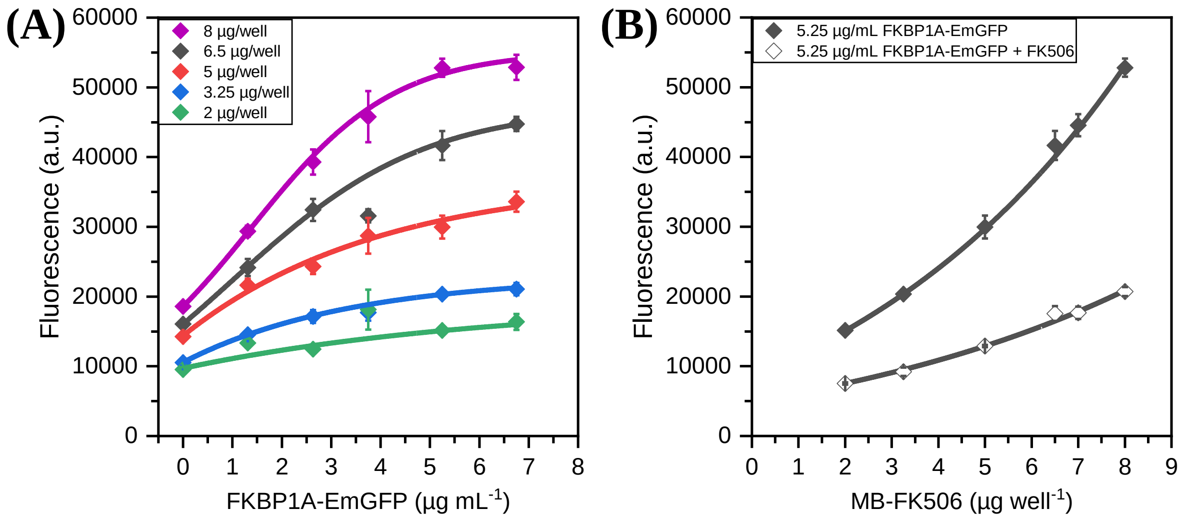

Figure S7. (A) Dilution curve for the FKBP1A-EmGFP as a function of the amount of MB-FK506 (2 – 8 µg/well). **(B)** Dilution curve for MB-FK506 as a function of the amount of FKBP1A-EmGFP (5.25 µg/mL) in the absence (black symbols, *n* = 3) and in the presence (opened symbols, *n* = 3) of 50 µg/mL of FK506. Results are shown as mean signals ± the standard error of the mean (*n* = 3).

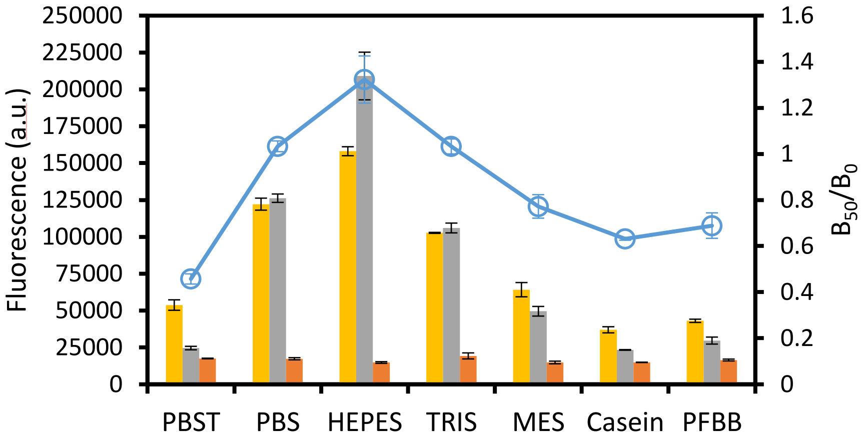

Figure S8. Effect of the buffer composition on the B_{50}/B_0 ratio (open symbols). The yellow bars correspond to the fluorescence signal in the absence of FK506 (B_0), the grey bars correspond to the signal in the presence of 50 ng/mL FK506 (B_{50}), and the orange bars to the background signal of the MB-FK506. PBST: phosphate buffer (10 mM, pH 7.4) with T20 (0.05%); PBS: phosphate buffer (10 mM, pH 7.4); HEPES: 4-(2-hydroxyethyl)-1-piperazineethanesulfonic acid (10 mM, pH 7.5); TRIS: 2-amino-2-(hydroxymethyl)propane-1,3-diol (10 mM, pH 8.0); MES: 2-(*N*-morpholino)ethanesulfonic acid buffer (10 mM, pH 4.7); Casein: Blocker™ Casein (1%, w/v) in PBS; PFBB: Pierce protein free (PBS) blocking buffer. Results are shown as mean signals ± the standard error of the mean (*n* = 3).

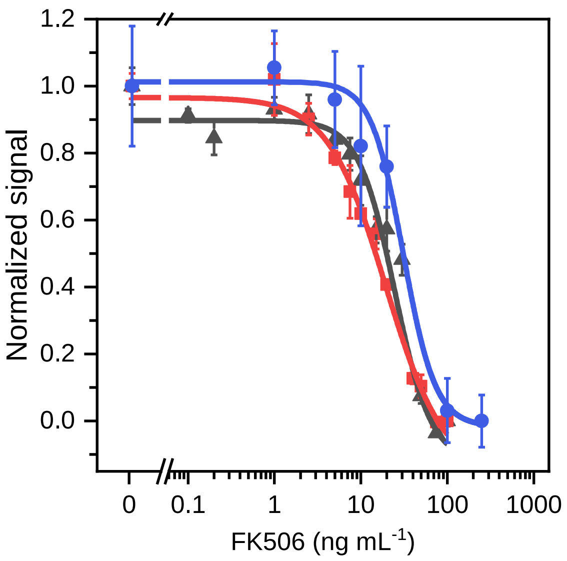

Figure S9. Optimization of the incubation time of FKBP1A-EmGFP with the analyte, FK506. Calibration plots corresponding to 15 min (black ▲), 25 min (red ■) and 35 min (blue ●), followed by 15 min incubation with MB-FK506. Results are shown as mean signals ± the standard error of the mean (*n* = 3).

Table S4. Performance of the fluororeceptor-based bioassay for quantification of FK506 in whole blood samples (*n* = 3).

Nominal concentration (ng mL^{-1})	Measured concentration (ng mL^{-1})	RSD (%)	Recovery (%)
10	10	5	104
13	14	6	109
16	15	13	95
20	18	9	87

**RSD, relative standard deviation (*n = 3*).*

Table S5. Comparative summary of analytical methods reported for the analysis of FK506 in biological samples.

Method	Sample material	Sample treatment	LOD/LOQ (ng mL^{-1})	DR (ng mL^{-1})	Ref./Manufactor
ACMIA	WB	FK506-carbamate + sonication lysis	0.7/1	1–30	Siemens (Dimension)
CEDIA	WB	PP: MeOH + $ZnSO_4$	-/2	2–30	Thermo Scientific
CMIA	WB	PP: MeOH + $ZnSO_4$	0.3/2	2–30	Abbott (ARCHITECT)
ECLIA	WB	PP: MeOH + $ZnSO_4$	0.5/1	0.5–40	Roche (Elecsys®)
EMIT	WB	PP: MeOH + $CuSO_4$	-/2	2–30	Siemens (EMIT 2000)
QMS	WB	PP: MeOH + $ZnSO_4$	-/0.9	1–30	Thermo Scientific
TRFIA	WB	Incubation 75 ºC, 15 min: Tris-HCl, urea, protease, T20, pH 8	0.57/-	1.1–28.6	9
FIA	Buffer	-	0.08/-	0.15–2	10
TIRF	Buffer	-	0.11/0.57	-	11
ECIS	S	-	0.17/-	1–30	12
ACMIA	WB	-	-/3	3–31.4	13
Paper ELISA	WB	-	1/-	-	14
CBS-MS/MS	WB	PP: $ZnSO_4$ (0.1 M):AcN:H_2O (60:30:10, v/v)	-/1	1–50	15
PS-MS/MS	DBS	Extraction: MeOH/$CHCl_3$/NaOAc (40/60/0.1, v/v)	0.2/1.5	1.5–30	16

Method	Sample material	Sample treatment	LOD/LOQ (ng mL^{-1})	DR (ng mL^{-1})	Ref./Manufactor
SPME-MOI-MS/MS	WB	PP: AcN/$ZnSO_4$ (0.1 M)/H_2O (30/60/10, v/v)	-/1	1–50	17
LC-MS/MS	DBS (8 mm)	PP: AcN/$ZnSO_4$	-	1.2–40	18
LC-MS/MS	WB	1) Centrifugation<br>2) MEPS on-line (C_8)	0.15/0.5	0.5–50	19
LC-MS/MS	DBS (6 mm)	1) PP: MeOH and sonication<br>2) LLE: *tert*-butil eter	0.25/1	1–80	20
LC-MS/MS	Saliva	1) Sonication<br>2) PP: AcN	-/0.01	0.01–1.6	21
Fluorescent receptor-based assay	WB	PP: MeOH and sonication	3/-	5–70	This work

ACMIA: antibody conjugated magnetic immunoassay; CBS: coated blade spray; CEDIA: cloned enzyme donor immunoassay; CMIA: chemiluminescent microparticle immunoassay; DBS: dried blood spot; DR: dynamic range; ECIS: electrochemical immunosensors; ECLIA: electrochemiluminescence immunoassay; EMIT: Enzyme multiplied immunoassay technique; FIA: fluorescence immunoassay; LC: liquid chromatography; LLE: liquid-liquid extraction; LOD: limit of detection; LOQ: limit of quantification; MEPS: micro extraction by packed sorbent; MOI: microfluidic open interface; MS/MS: tandem mass spectrometry; PP: protein precipitation; PS: paper spray; QMS: Quantitative Microsphere System; S: serum; SPME: solid-phase microextraction; TIRF: total internal reflection fluorescence; TRFIA: time-resolved fluorescence immunoassay; WB: whole blood.

Table S6. Administered doses of FK506 to the analyzed organ transplanted patients.

Patient	Administered drug	Dose/24 h
HCUV1	Advagraf	11 mg
HCUV2	Advagraf	6 mg
HCUV3	Envarsus	4 mg
HCUV4	Envarsus	6 mg
HCUV5	Advagraf	3.5 mg
HCUV6	Advagraf	6.5 mg
HCUV7	Advagraf	2 mg

References

(1) Sauerbrey, G. Verwendung von Schwingquarzen zur Wägung dünner Schichten und zur Mikrowägung. *Z. Physik* **1959**, *155* (2), 206–222. https://doi.org/10.1007/BF01337937.

(2) Voinova, M. V.; Rodahl, M.; Jonson, M.; Kasemo, B. Viscoelastic Acoustic Response of Layered Polymer Films at Fluid-Solid Interfaces: Continuum Mechanics Approach. *Phys. Scr.* **1999**, *59* (5), 391. https://doi.org/10.1238/Physica.Regular.059a00391.

(3) Gambinossi, F.; Banchelli, M.; Durand, A.; Berti, D.; Brown, T.; Caminati, G.; Baglioni, P. Modulation of Density and Orientation of Amphiphilic DNA Anchored to Phospholipid Membranes. I. Supported Lipid Bilayers. *J. Phys. Chem. B* **2010**, *114* (21), 7338–7347. https://doi.org/10.1021/jp100730x.

(4) Gambinossi, F.; Lorenzelli, L.; Baglioni, P.; Caminati, G. Silicon Oxide Surface Functionalization by Self-Assembled Nanolayers for Microcantilever Transducers. *Colloids Surf. A Physicochem. Eng. Asp* **2008**, *321* (1), 87–93. https://doi.org/10.1016/j.colsurfa.2008.01.030.

(5) Baltus, R. E.; Carmon, K. S.; Luck, L. A. Quartz Crystal Microbalance (QCM) with Immobilized Protein Receptors: Comparison of Response to Ligand Binding for Direct Protein Immobilization and Protein Attachment via Disulfide Linker. *Langmuir* **2007**, *23* (7), 3880–3885. https://doi.org/10.1021/la0628468.

(6) Ueda, E. K. M.; Gout, P. W.; Morganti, L. Current and Prospective Applications of Metal Ion–Protein Binding. *J. Chromatogr. A* **2003**, *988* (1), 1–23. https://doi.org/10.1016/S0021-9673(02)02057-5.

(7) Raghunath, G.; Dyer, R. B. Kinetics of Histidine-Tagged Protein Association to Nickel-Decorated Liposome Surfaces. *Langmuir* **2019**, *35* (38), 12550–12561. https://doi.org/10.1021/acs.langmuir.9b01700.

(8) Nye, J. A.; Groves, J. T. Kinetic Control of Histidine-Tagged Protein Surface Density on Supported Lipid Bilayers. *Langmuir* **2008**, *24* (8), 4145–4149. https://doi.org/10.1021/la703788h.

(9) Wu, F.-B.; Yang, Y.-Y.; Wang, X.-B.; Wang, Z.; Zhang, W.-W.; Liu, Z.-Y.; Qian, Y.-Q. A Sample Processing Method for Immunoassay of Whole Blood Tacrolimus. *Anal. Biochem.* **2019**, *576*, 13–19. https://doi.org/10.1016/j.ab.2019.04.006.

(10) Salis, F.; Descalzo, A. B.; Benito-Peña, E.; Moreno-Bondi, M. C.; Orellana, G. Highly Fluorescent Magnetic Nanobeads with a Remarkable Stokes Shift as Labels for Enhanced Detection in Immunoassays. *Small* **2018**, *14* (20), 1703810. https://doi.org/10.1002/smll.201703810.

(11) Berrettoni, C.; Berneschi, S.; Bernini, R.; Giannetti, A.; Grimaldi, I. A.; Persichetti, G.; Testa, G.; Tombelli, S.; Trono, C.; Baldini, F. Optical Monitoring of Therapeutic Drugs with a Novel Fluorescence- Based POCT Device. *Procedia Eng.* **2014**, *87*, 392–395. https://doi.org/10.1016/j.proeng.2014.11.732.

(12) Zhang, Z.; Zhang, Y.; Yu, H.; Rong, S.; Gao, H.; Meng, L.; Dai, J.; Pan, H.; Chang, D. Spherical Carrier Amplification Strategy for Electrochemical Immunosensor Based on Polystyrene-Gold Nanorods @L-Cysteine/MoS2 for

Determination of Tacrolimus. *Talanta* **2020**, *220*, 121321. https://doi.org/10.1016/j.talanta.2020.121321.

(13) Tempestilli, M.; Di Stasio, E.; Basile, M. R.; Elisei, F.; Antonini, M.; Ettorre, G. M.; Iappelli, M.; Pucillo, L. P. Low Plasma Concentrations of Albumin Influence the Affinity Column–Mediated Immunoassay Method for the Measurement of Tacrolimus in Blood During the Early Period After Liver Transplantation. *Ther. Drug Monit.* **2013**, *35* (1), 96–100. https://doi.org/10.1097/FTD.0b013e318279dfd2.

(14) Lantigua, D.; Trimper, J.; Unal, B.; Camci-Unal, G. A New Paper-Based Biosensor for Therapeutic Drug Monitoring. *Lab Chip* **2021**, *21* (17), 3289–3297. https://doi.org/10.1039/D1LC00473E.

(15) Rickert, D. A.; Gómez-Ríos, G. A.; Nazdrajić, E.; Tascon, M.; Kulasingam, V.; Pawliszyn, J. B. Evaluation of a Coated Blade Spray-Tandem Mass Spectrometry Assay as a New Tool for the Determination of Immunosuppressive Drugs in Whole Blood. *Anal. Bioanal. Chem.* **2020**, *412* (21), 5067–5076. https://doi.org/10.1007/s00216-019-02367-z.

(16) Shi, R.-Z.; El Gierari, E. T. M.; Manicke, N. E.; Faix, J. D. Rapid Measurement of Tacrolimus in Whole Blood by Paper Spray-Tandem Mass Spectrometry (PS-MS/MS). *Clin. Chim. Acta* **2015**, *441*, 99–104. https://doi.org/10.1016/j.cca.2014.12.022.

(17) Tascon, M.; Alam, Md. N.; Gómez-Ríos, G. A.; Pawliszyn, J. Development of a Microfluidic Open Interface with Flow Isolated Desorption Volume for the Direct Coupling of SPME Devices to Mass Spectrometry. *Anal. Chem.* **2018**, *90* (4), 2631–2638. https://doi.org/10.1021/acs.analchem.7b04295.

(18) Sadilkova, K.; Busby, B.; Dickerson, J. A.; Rutledge, J. C.; Jack, R. M. Clinical Validation and Implementation of a Multiplexed Immunosuppressant Assay in Dried Blood Spots by LC–MS/MS. *Clin. Chim. Acta* **2013**, *421*, 152–156. https://doi.org/10.1016/j.cca.2013.02.009.

(19) Said, R.; Pohanka, A.; Abdel-Rehim, M.; Beck, O. Determination of Four Immunosuppressive Drugs in Whole Blood Using MEPS and LC–MS/MS Allowing Automated Sample Work-up and Analysis. *J. Chromatogr. B* **2012**, *897*, 42–49. https://doi.org/10.1016/j.jchromb.2012.04.006.

(20) Li, Q.; Cao, D.; Huang, Y.; Xu, H.; Yu, C.; Li, Z. Development and Validation of a Sensitive LC-MS/MS Method for Determination of Tacrolimus on Dried Blood Spots. *Biomed. Chromatogr.* **2013**, *27* (3), 327–334. https://doi.org/10.1002/bmc.2795.

(21) Ghareeb, M.; Akhlaghi, F. Development and Validation of a Sensitive and Selective LC–MS/MS Method for Determination of Tacrolimus in Oral Fluids. *J. Chromatogr. B* **2016**, *1038*, 136–141. https://doi.org/10.1016/j.jchromb.2016.10.008.
